# HPV E6/E7, O-GlcNAcylation and mitochondrial remodeling in cervical cancer: mechanistic links and translational opportunities

**DOI:** 10.3389/fmolb.2026.1919738

**Published:** 2026-10-30

**Authors:** Yingying Zhang, YiMei Tao, Huirong He, Ping Li, Minbin Chen

**Affiliations:** 1 Department of Radiotherapy and Oncology, Gusu School, Nanjing Medical University, The First People’s Hospital of Kunshan, Kunshan, China; 2 Department of Radiotherapy and Oncology, First People’s Hospital of Kunshan, Kunshan, China

**Keywords:** cervical cancer, hexosamine biosynthetic pathway, HPV E6/E7, metabolic reprogramming, mitochondrial metabolism, O-GlcNAcylation, tumor microenvironment

## Abstract

Persistent high-risk human papillomavirus (HPV) infection initiates cervical carcinogenesis, while progression and treatment resistance also involve metabolic adaptation. O-GlcNAcylation is a reversible post-translational modification (PTM) controlled by O-GlcNAc transferase (OGT) and O-GlcNAcase (OGA), with UDP-GlcNAc generated by the hexosamine biosynthetic pathway (HBP) serving as the donor substrate. This review evaluates evidence linking HPV E6/E7-associated metabolic demand, HBP flux, O-GlcNAcylation, and mitochondrial function. We distinguish observations made under untreated conditions from acute treatment responses, identify whether validated resistant models were used, and examine HPV genotype as a source of heterogeneity. Cervical cancer studies support links between O-GlcNAcylation and pathways involved in growth and survival, but no mitochondrial O-GlcNAcylation site has been causally validated in HPV-positive cervical cancer. We therefore grade evidence by model, temporal context, and causal depth and place the proposed relationships within a broader PTM network. This synthesis separates findings obtained in cervical cancer from mechanisms inferred from other systems and identifies the main gaps limiting biomarker and therapeutic development.

## Highlights

### FACT

- Persistent high-risk HPV infection is the principal initiating event in cervical carcinogenesis, whereas sustained E6/E7 expression drives malignant transformation and tumor maintenance.
- UDP-GlcNAc generated by the HBP links glucose, glutamine, acetyl-CoA, and nucleotide metabolism to O-GlcNAcylation.
- O-GlcNAcylation has been linked to oncogenic signaling and survival under stress, but evidence for mitochondrial regulation comes mainly from other models. No mitochondrial O-GlcNAcylation site or acquired resistance mechanism has been validated in cervical cancer models expressing HPV E6/E7.

### Open questions

- How do HPV genotype and variant lineage, histology, disease stage, and temporal context, including untreated HPV-associated states, acute treatment responses, and durable resistance, alter OGT/OGA activity, HBP flux, and mitochondrial O-GlcNAcylation?
- Which O-GlcNAcylation events on specific proteins causally alter ATP production, ROS levels, membrane potential, the apoptotic threshold, or treatment response, rather than merely reflect increased glucose demand, hypoxia, or injury caused by treatment?
- Can biomarkers based on O-GlcNAcylation be reliably detected in tissue, blood, cervicovaginal fluid, or extracellular vesicles and used for early detection, patient stratification, or treatment monitoring?

## 1 Introduction

Cervical cancer is one of the most common malignancies in women worldwide. According to GLOBOCAN 2022, an estimated 662,301 new cases of cervical cancer and 348,874 deaths occurred worldwide in 2022 (Bray et al., 2024). Persistent infection with high-risk HPV is a key driver of malignant transformation of the cervical epithelium, and sustained expression of the viral E6/E7 oncoproteins plays a central oncogenic role (Zhang Y. et al., 2025). However, HPV positivity alone cannot fully explain why some lesions persist, become invasive, metastasize, or develop treatment resistance. Many HPV-positive lesions do not progress to invasive cancer. By contrast, established cervical cancer cells must sustain proliferation, resist oxidative stress, evade apoptosis, and tolerate therapeutic pressure (Hu et al., 2022; Boroughs and DeBerardinis, 2015; Ma et al., 2025). This contrast indicates that metabolic and mitochondrial adaptations, including altered glycolysis, antioxidant defense, membrane lipid biogenesis, and mitochondrial function, should be considered alongside viral oncogene activity during cervical cancer evolution (Li and Sui, 2021).

During malignant progression, protein glycosylation is increasingly recognized as a mechanism that links extracellular stimuli, intracellular metabolic state, and signaling output. Classical N-glycosylation mainly participates in protein folding, membrane trafficking, receptor stability, and cell-cell communication (He et al., 2024). O-GlcNAcylation acts more like a dynamic signaling regulator than a traditional structural glycan decoration (Le Minh et al., 2023; Lu et al., 2022; Slawson and Hart, 2011).

O-GlcNAcylation is the reversible addition of a single N-acetylglucosamine moiety to serine or threonine residues on proteins (Le Minh et al., 2023). O-GlcNAc transferase (OGT) adds O-GlcNAc, whereas O-GlcNAcase (OGA) removes it. The donor substrate, UDP-GlcNAc is generated by the hexosamine biosynthetic pathway (HBP) from inputs derived from glucose, glutamine, lipids, and nucleotides (Paneque et al., 2023). By coupling these metabolic inputs to changes in protein stability, localization, and activity, O-GlcNAc cycling can report nutrient availability, biosynthetic demand, and cellular stress without acting as the sole upstream controller of those states (Le Minh et al., 2023; Paneque et al., 2023; Gawlowski et al., 2012).

Cervical cancer models show variable mitochondrial oxidative activity rather than a consistent reduction in OXPHOS (Li and Sui, 2021; Riera Leal et al., 2020). In HeLa and SiHa cells and cervical tumor tissues, formation of a mitochondrial complex containing CDC28 protein kinase regulatory subunit 2 (CKS2) and single-stranded DNA-binding protein 1 (SSBP1) was associated with an OXPHOS-related transcriptional program and chemoradioresistance (Jonsson et al., 2019). Mitochondria therefore remain active contributors to ATP production, ROS signaling, biosynthetic precursor supply, apoptosis control, innate immune signaling, and stress responses (Zong et al., 2016; O'Malley et al., 2020; Kuo et al., 2022).

Accumulating evidence from diverse experimental systems indicates that O-GlcNAcylation can modify proteins involved in mitochondrial dynamics, respiration, metabolism, and apoptosis (Gawlowski et al., 2012; Qiu et al., 2024). A single study in a cardiac model reported rapid changes in OXPHOS, Complex I activity, and ROS release after manipulation of intramitochondrial O-GlcNAcylation (Dontaine et al., 2022). Without independent replication or validation in cervical cancer, these findings constitute preliminary evidence from another model rather than an established mechanism. An integrated cervical cancer-specific framework remains lacking, particularly at the level of defined mitochondrial substrates and modification sites.

Accordingly, this review evaluates a proposed connection among HPV oncogenic pressure, HBP metabolic flux, O-GlcNAcylation, and mitochondrial adaptation in relation to the source and strength of the supporting evidence (Figure 1). Evidence is considered separately according to HPV genotype, experimental model, treatment status, and causal depth. Persistent HPV E6/E7 expression is associated with glycolytic and other metabolic programs (Hu et al., 2022; Arizmendi-Izazaga et al., 2024; Gore et al., 2025), but direct effects on ATP production, ROS handling, the partitioning of TCA-cycle carbon between oxidation, anaplerosis, and biosynthesis, mitochondrial membrane potential, and the apoptotic threshold are not uniformly established (Arizmendi-Izazaga et al., 2024; Gore et al., 2025). Links supported only by non-cervical models are treated as hypotheses requiring validation in HPV-positive cervical cancer cells, patient-derived models, and clinical samples (Ma et al., 2025; Klein et al., 2020).

**FIGURE 1:**
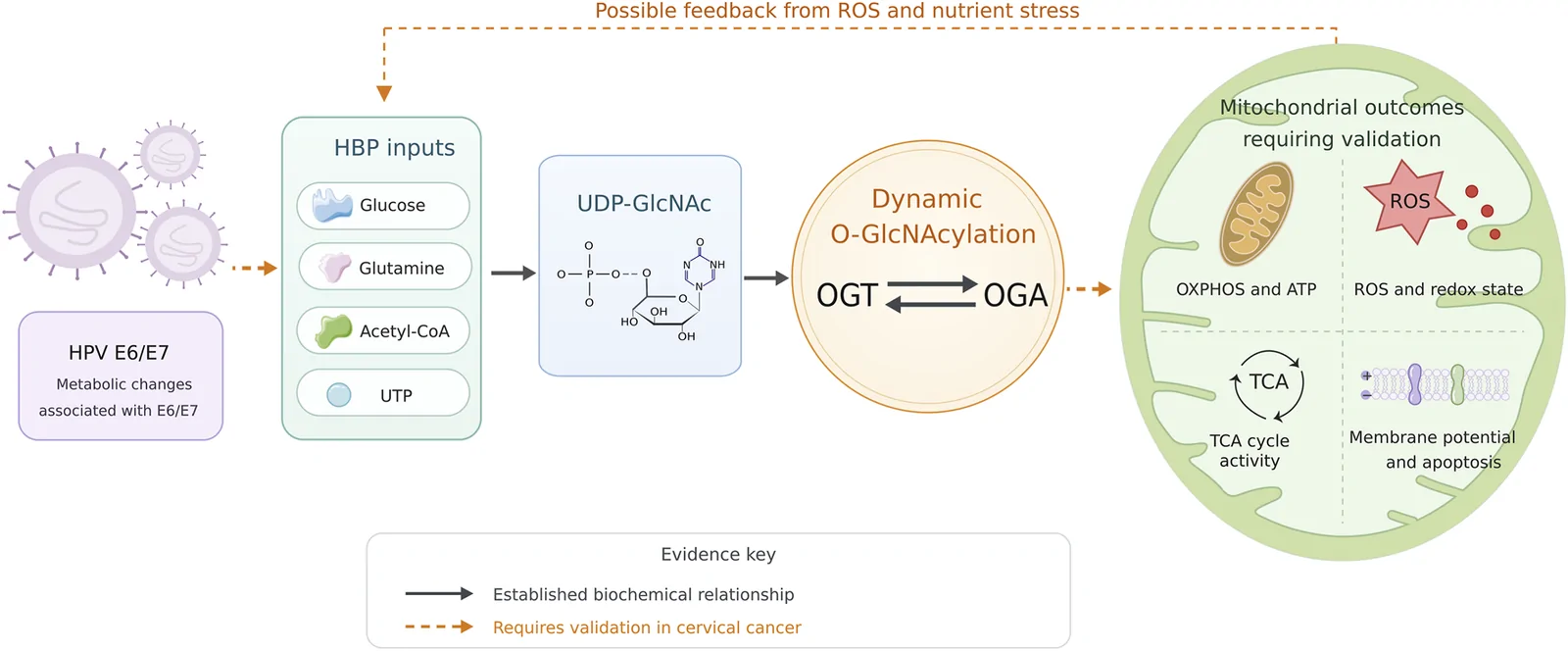
Proposed relationship between HPV E6/E7, O-GlcNAcylation, and mitochondrial outcomes. The hexosamine biosynthetic pathway generates UDP-GlcNAc, which supports modification by OGT. OGA removes this modification. Candidate mitochondrial outcomes include OXPHOS, ATP production, ROS and redox state, TCA cycle activity, membrane potential, and apoptosis. Black solid arrows indicate established biochemical relationships. Orange dashed arrows indicate relationships requiring validation in cervical cancer, including possible feedback from ROS and nutrient stress.

### 1.1 Literature search and evidence selection approach

PubMed was searched from database inception to 1 September 2026. Topic-specific Boolean search blocks covered: (i) cervical cancer and O-GlcNAcylation/HBP; (ii) HPV E6/E7 and O-GlcNAcylation/OGT; (iii) O-GlcNAcylation and mitochondrial function or dynamics, including OXPHOS, respiration, ROS/redox regulation, membrane potential, apoptosis, Drp1/DNM1L, OPA1, MFN1/2, HK2, and VDAC1; and (iv) cervical cancer-specific mechanisms of treatment resistance and the tumor microenvironment. MeSH and Title/Abstract terms were combined where appropriate.

Reference lists of relevant primary studies and reviews were also screened. Peer-reviewed primary studies conducted in cervical cancer models or clinical samples were prioritized, with HPV-positive models preferred where available. When cervical cancer-specific evidence was insufficient, studies from other tumor or non-tumor models were included and identified as evidence from other models. As this was a narrative review, no formal meta-analysis or assessment of risk of bias was performed.

## 2 HPV E6/E7 associated metabolic demand and O-GlcNAcylation

### 2.1 Sustained HPV E6/E7 expression increases metabolic demand in cervical cancer cells

High-risk HPV16 and HPV18 are the predominant oncogenic genotypes in cervical cancer. Persistent E6/E7 expression disrupts cell-cycle control, apoptosis, and metabolic homeostasis (Pal and Kundu, 2019; Bhattacharjee et al., 2022). E6 promotes p53 degradation mainly through an E6AP-dependent mechanism, whereas E7 binds pRB family proteins through its LXCXE motif and releases G1/S checkpoint control (Bhattacharjee et al., 2022; Vats et al., 2021). E6/E7 also engage c-Myc, HIF-1α, PI3K/Akt, PKM2, and E2F-related programs that regulate glycolysis, glutamine utilization, nucleotide synthesis, and adaptation to oxidative stress (Arizmendi-Izazaga et al., 2024; Vats et al., 2021; Arizmendi-Izazaga et al., 2021). These observations establish a link between viral oncogene activity and host metabolism. However, the available evidence does not establish a mechanism linking E6/E7 expression, the HBP, and O-GlcNAcylation that is shared across HPV genotypes.

E6 and E7 show marked structural plasticity and interact with multiple host targets. E6 contains conserved zinc-binding domains and a C-terminal postsynaptic density protein 95/discs large/zonula occludens 1 (PDZ)-binding motif, enabling it to act as an adaptor in multiprotein complexes (Vats et al., 2021; Yoshimatsu et al., 2017). E7 contains CR1, CR2, and CR3 regions (Moody and Laimins, 2010). Its N-terminal LXCXE motif mediates pRB binding, whereas its C-terminal zinc-finger structure contributes to dimerization and interactions with non-canonical targets (Zhang et al., 2006). These short linear motifs and flexible structures connect E6/E7 to chromatin modification, transcription, cell-cycle progression, and metabolic regulatory networks (Glavina et al., 2022).

Notably, HPV E6 can elevate OGT expression and O-GlcNAcylation, and the E6/E7 oncoproteins interact with host metabolic networks (Zeng et al., 2016; Peng et al., 2021; Kim et al., 2016). E6/E7-associated proliferation is accompanied by increased glycolysis and biosynthetic demand (Hu et al., 2022; Arizmendi-Izazaga et al., 2024; Vats et al., 2021; Arizmendi-Izazaga et al., 2021). Greater glycolytic and biosynthetic activity can alter HBP substrate availability. However, the available evidence does not demonstrate that O-GlcNAcylation initiates metabolic reprogramming. We therefore position HBP/OGT/OGA changes as a potentially responsive and feedback-regulated layer that requires direct flux, substrate, and rescue experiments in HPV-positive models (Jiménez-Castillo et al., 2022).

### 2.2 HPV genotype and variant heterogeneity

The metabolic effects of HPV16 and HPV18 should not be assumed to be interchangeable. Their E6/E7 proteins differ in sequence, expression control, splice products, and affinity for selected host partners (Vats et al., 2021). A common comparison between SiHa (HPV16-positive squamous carcinoma) and HeLa (HPV18-positive adenocarcinoma) cells also confounds viral genotype with histology, integration pattern, viral copy number, and host genomic background (Pappa et al., 2021; Sun et al., 2022).

Studies of HPV16 E6/E7 and HPV16 variants have implicated c-Myc and HIF-1α-related metabolic programs (Hu et al., 2022; Arizmendi-Izazaga et al., 2024; Arizmendi-Izazaga et al., 2021). Comparable mechanistic evidence for HPV18 and other high-risk genotypes remains limited. Available evidence does not support assigning a consistent direction or magnitude to the effects of HPV16 versus HPV18 E6/E7 on c-Myc or HIF-1α. Within the literature included in this review, we identified no comparison of matched HPV16 and HPV18 models that quantified HBP flux, UDP-GlcNAc abundance, OGT/OGA activity, or site-specific O-GlcNAcylation. The available metabolomic comparison showed features of Warburg metabolism in both SiHa and HeLa cells but different nicotinamide and redox profiles. SiHa cells showed accumulation of NAD, NADH, and several glutathione pathway metabolites, whereas HeLa cells showed increased nicotinamide riboside and cysteine (Pappa et al., 2021). Because SiHa and HeLa are not isogenic, the observed differences cannot be assigned to HPV genotype alone. HPV genotype should therefore be treated as a prespecified stratification variable rather than an established determinant of HBP activity or O-GlcNAcylation. Isogenic keratinocyte or cervical organoid models expressing matched E6/E7 constructs from HPV16, HPV18, and major variant lineages are needed to resolve this question.

### 2.3 HBP supplies UDP-GlcNAc for O-GlcNAcylation

Unlike classical glycosylation in the secretory pathway, O-GlcNAcylation occurs on proteins in the nucleus, cytosol, and mitochondrial matrix (Yu et al., 2022) and can respond rapidly to nutrients, oxidative stress, viral infection, and therapeutic stress (Shi Y. et al., 2022; Ma and Hart, 2014). At individual proteins and sites, it may cooperate or compete with phosphorylation (Hart et al., 2011).

O-GlcNAcylation operates within a broader PTM network in HPV-positive cells. Phosphorylation, acetylation, and other lysine acylations, including lactylation, also regulate signaling, chromatin state, and metabolism. O-GlcNAcylation can compete or cooperate with phosphorylation at serine/threonine residues, while lysine acetylation and lactylation can couple metabolite availability to enzyme activity and transcriptional programs (Hart et al., 2011; Feng et al., 2024; Zhao et al., 2010; Zhang et al., 2019). This review focuses on O-GlcNAcylation because the HBP supplies its donor substrate, UDP-GlcNAc.

The HBP uses glucose, glutamine, acetyl-CoA, and UTP to generate UDP-GlcNAc, the donor substrate for O-GlcNAcylation (Kim Y. H. et al., 2018; Ye et al., 2020). HBP flux is therefore sensitive to the availability of these metabolic inputs. UDP-GlcNAc also inhibits glutamine fructose-6-phosphate amidotransferase 1 (GFAT1), encoded by GFPT1, through feedback regulation (Kim Y. H. et al., 2018; Ye et al., 2020; Ruegenberg et al., 2020). In HPV-associated cervical cancer, E6/E7-driven proliferation and stress may change nutrient demand and substrate availability, thereby altering HBP flux and O-GlcNAcylation. However, this responsiveness does not establish O-GlcNAcylation as the initiating cause of metabolic reprogramming, because phosphorylation and acetylation networks also respond to metabolic and signaling states (Hart et al., 2011; Zhao et al., 2010). Testing this relationship in HPV-positive cervical cancer requires direct measurement of HBP flux and substrate-specific O-GlcNAcylation, together with perturbation and rescue experiments linked to the relevant metabolic phenotype.

### 2.4 Regulation of O-GlcNAcylation by OGT/OGA across temporal contexts

O-GlcNAcylation of individual proteins is regulated by the opposing activities of OGT and OGA and varies across cellular compartments and over time (Boroughs and DeBerardinis, 2015; Paneque et al., 2023). OGT substrate selection does not rely on a fixed kinase-like recognition motif (Chong et al., 2023). The N-terminal tetratricopeptide repeat domain of OGT regulates substrate recognition and subcellular localization through protein-protein interactions (Lubas and Hanover, 2000). OGT can therefore modify different substrates according to cell type, nutrient status, subcellular compartment, and stress conditions (Hart et al., 2011). In high-risk HPV-associated cervical cancer, a central mechanistic question is which transcription factors, metabolic enzymes, mitochondrial proteins, or HPV-associated host factors are functionally targeted by OGT (Zeng et al., 2016; Kim et al., 2016).

Because O-GlcNAcylation changes with nutrient and stress conditions, its interpretation depends on sampling time and exposure history. Measurements from untreated models, acute treatment responses (Wang et al., 2021), and durable resistance models represent distinct temporal contexts and should not be interpreted interchangeably. In HeLa cells, DNA damage acutely recruited OGT to damage sites (Chen and Yu, 2016), whereas HPV E6 increased OGT mRNA and protein in transduced cells (Zeng et al., 2016). Stress-induced increases in O-GlcNAcylation can also be transient in other experimental systems (Kátai et al., 2016). A durable resistant phenotype requires a validated resistant model and should persist after drug withdrawal or recovery where feasible (Liu et al., 2018; Ravindran Menon et al., 2023).

## 3 O-GlcNAcylation in signaling networks associated with HPV

Persistent E6/E7 expression is associated with activation of PI3K/Akt/mTOR, NF-κB, c-Myc, HIF-1α, and related networks controlling growth, inflammation, and metabolism (Arizmendi-Izazaga et al., 2024; Vats et al., 2021; Arizmendi-Izazaga et al., 2021). Multiple PTMs, including phosphorylation and acetylation, regulate these pathways, while O-GlcNAcylation represents one interacting layer rather than an exclusive controller (Hart et al., 2011; Zhao et al., 2010). Within these networks, OGT was reported to increase E6/E7 promoter activity in HeLa cells, and depletion of host cell factor 1 (HCF-1) reduced the increase in promoter activity (Kim et al., 2016) (Table 1). Available studies in cervical cancer support selected associations involving O-GlcNAcylation. Table 1 summarizes the model source and strength of evidence for each relationship.

**TABLE 1:** Evidence levels for mechanisms involving O-GlcNAcylation that are relevant to cervical cancer and mitochondrial phenotypes.

Protein or pathway	Tier	Model	Perturbation and timing	Mitochondrial evidence	Reported outcome
RACK1 S122 (Zhang et al., 2023)	T1B	SiHa, HeLa and MS751; HEK293T for S122A testing	circVPRBP perturbation; time NR	Not assessed	RACK1 stability and lymph node metastasis
STAT1 Ser727 (Wu et al., 2026)	T1A	CaSki and THP-1-derived macrophage coculture	OGT overexpression and depletion, STAT1 knockout, and Ser727 mutation; time NR	Not assessed	Macrophage polarization and phagocytosis
OGT/HCF-1 regulation of E6/E7^33^	T1B	HeLa and SiHa; HeLa xenograft	Low or high glucose, duration NR; stable OGT depletion; xenograft assessed at 8 weeks	Not assessed	E6/E7 expression and tumor growth
IGF1R/PI3K/Akt (Jiménez-Castillo et al., 2022)	T1B	CaSki	Thiamet G for 24 h; glucosamine for 6 h; IGF-1 for 10 min	Not assessed	Proliferation, migration and survival
NF-κB p65 Thr322 and Thr352 regulation of CXCR4 (Ali et al., 2017)	T1A	HeLa; mouse lung tissue	p65 site mutants; stable OGT depletion; DON or Thiamet G for 24 h; lung tissue assessed at 3 months	Not assessed	CXCR4 expression; lung metastasis after OGT depletion
OGT/EMT-associated networks (Gao et al., 2018)	T1B	HeLa and CaSki	OGT knockdown or overexpression; migration assessed at 48 h and invasion at 18–24 h	Not assessed	EMT-marker expression, migration and invasion
GFPT1/PTEN (Li et al., 2022)	T1B	HeLa and SiHa	GFPT1 knockdown or overexpression; L-DON duration NR	Not assessed	PTEN degradation and proliferation
OGT/LXR/sCLU (Kim et al., 2018b)	T1B	HeLa	Stable OGT depletion before cisplatin; cisplatin for 24 h	Not assessed	Increased cisplatin sensitivity after OGT depletion
AMPK O-GlcNAcylation (Kim et al., 2019)	T1B	HeLa	Metformin for 24 h	Not assessed	Cell cycle arrest and apoptosis
Raptor T700/mTORC1 (Xu et al., 2023)	T3	HEK293T	Glucose starvation for 2 h followed by glucose stimulation	Not assessed	mTORC1 activation
c-Myc S415/PDK2 (Wang et al., 2024a)	T3	HT-29 and HCT116	Stable OGT or c-Myc perturbation; WT/S415A rescue	Direct	Suppressed mitochondrial pyruvate oxidation and promoted tumor growth
HIF-1α/GLUT1 (Ferrer et al., 2014)	T3	MDA-MB-231 and MCF-7	Stable OGT perturbation; NButGT for 48 h	Not assessed	Glycolysis and cell survival
PFK1 S529 (Yi et al., 2012)	T3	H1299, A549 and HEK293T	Hypoxia at 0.5% O_2_ for 24 h	Not assessed	Pentose phosphate pathway flux and tumor growth
ACLY S979 (Liu et al., 2024)	T3	A549 and other tumor models	Glucose deprivation for 6 h; EGF sampled at 0, 6 and 48 h	Not assessed	Lipogenesis and proliferation
HK2 S478 (Panpan et al., 2025)	T3	H1975 cells and xenografts	HK2 knockdown and WT/S478V transfection for 48 h; xenograft assessed on day 28	Direct	Mitochondrial morphology, membrane potential, mitochondrial ROS and tumor growth
Drp1 (Akinbiyi et al., 2021)	T3	OGA-knockout MEFs; glioblastoma cells; HCT116	Stable OGA knockout in MEFs; assay time NR	Direct	Mitochondrial morphology and function
Drp1 T585/T586 (Park et al., 2021)	T3	SH-SY5Y cells and primary neurons	Single Aβ or PugNAc exposure; duration NR	Direct	Drp1 O-GlcNAcylation and mitochondrial fragmentation

Model annotations. SiHa and CaSki are HPV16-positive. HeLa is HPV18-positive. MS751 is HPV45-positive. These labels identify the models and do not imply genotype-specific causation. Evidence tiers are defined as follows. T1A denotes cervical cancer evidence with a functionally validated O-GlcNAcylation site. T1B denotes cervical cancer functional evidence without site validation. T2 denotes cervical cancer pathway or correlative evidence. T3 denotes evidence from other models. Direct mitochondrial evidence indicates that a mitochondrial endpoint was examined. Not assessed indicates that no such endpoint was examined. Abbreviations used only in the table: NR, not reported; WT, wild type; MEFs, mouse embryonic fibroblasts; DON, and L-DON, 6-diazo-5-oxo-L-norleucine; NButGT, and PugNAc, O-GlcNAcase inhibitors; Aβ, amyloid-β.

The PI3K/Akt/mTOR pathway regulates cell growth, glucose uptake, glycolysis, and biosynthesis (Chong et al., 2023; Xu et al., 2023; Jiang et al., 2020) (Table 1). Studies in other models link O-GlcNAcylation to Akt phosphorylation and identify Raptor O-GlcNAcylation as a regulator of mTORC1 signaling (Li et al., 2014; Ouyang et al., 2022; Liu and Ramakrishnan, 2021). In cervical cancer, increased O-GlcNAcylation has been associated with IGF1R/PI3K/Akt activation, proliferation, and invasion (Jiménez-Castillo et al., 2022) (Table 1). For NF-κB signaling, OGT depletion in HeLa cells reduced p65 O-GlcNAcylation and C-X-C motif chemokine receptor 4 (CXCR4) expression (Ali et al., 2017).

In cervical cancer models, HPV E6-associated increases in O-GlcNAcylation have been linked to c-Myc stabilization (Zeng et al., 2016). O-GlcNAcylation dependent regulation of c-Myc has also been reported in prostate and colorectal cancer models (Wang H. et al., 2024; Itkone et al., 2013). Evidence linking O-GlcNAcylation to HIF-1α comes from other models (Wang et al., 2023; Ferrer et al., 2014) (Table 1). HIF-1α activates a broad glycolytic transcriptional program that includes glucose transporters and multiple glycolytic enzymes. GLUT1, LDHA, and PDK1 are representative examples rather than an exhaustive target list (Wei et al., 2022; Semenza, 2012).

Figure 2 summarizes these signaling relationships and distinguishes cervical cancer evidence from evidence obtained in other models. The effects of these pathways on mitochondrial phenotypes in HPV-positive cervical cancer remain unresolved and are examined in Section 3.

**FIGURE 2:**
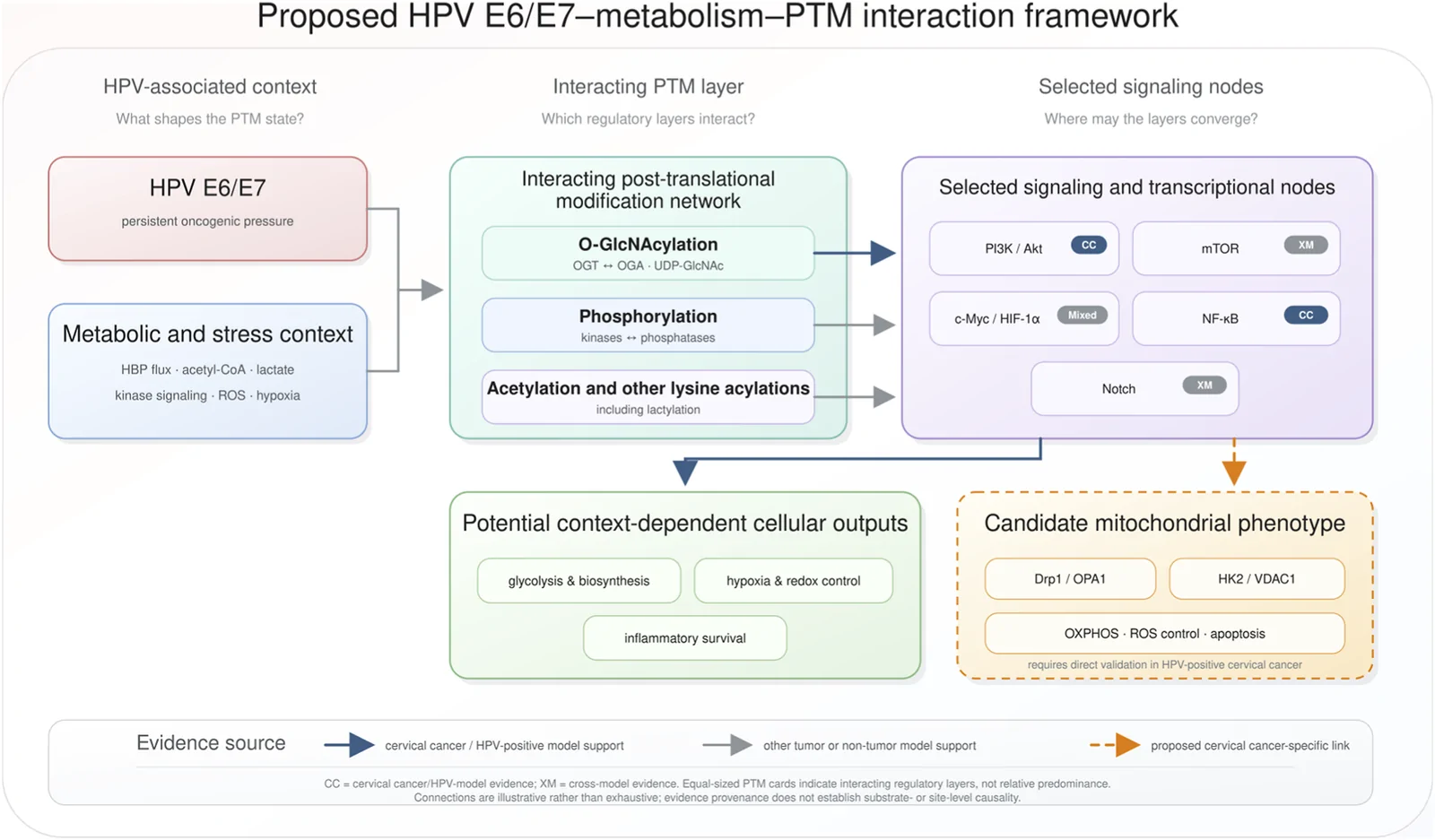
Proposed interactions between HPV E6/E7 associated metabolism and an interacting PTM network. Dark-blue solid arrows indicate relationships supported in cervical cancer or HPV-positive models; gray solid arrows indicate evidence from other tumor or non-tumor models; orange dashed arrows denote proposed cervical cancer-specific links. Nodes and arrows are illustrative rather than exhaustive and do not establish substrate-level causality.

## 4 O-GlcNAcylation regulates mitochondrial functional changes

### 4.1 Evidence boundaries for direct and putative effects of O-GlcNAcylation on mitochondrial function

Mitochondria integrate energy production, redox signaling, and apoptosis. In HPV-positive cervical cancer, persistent E6/E7 expression is associated with proliferative and metabolic programs; however, direct E6/E7-dependent changes in mitochondrial ATP production, ROS handling, membrane potential, and apoptotic thresholds have not been uniformly established (Arizmendi-Izazaga et al., 2024; Gore et al., 2025). The available evidence supports investigation of mitochondrial function in cervical cancer but does not establish mitochondrial O-GlcNAcylation as a causal mechanism. OXPHOS, ROS generated by the electron transport chain, membrane potential, mitochondrial outer membrane permeabilization, and cytochrome c release therefore represent functional readouts for testing the proposed role of O-GlcNAcylation (Klein et al., 2020).

Among the studies included in this review, one study directly manipulated intramitochondrial O-GlcNAcylation. Using an acute 30 min intervention in isolated cardiac mitochondria, the study reported changes in Complex I activity, respiratory capacity, and ROS release (Dontaine et al., 2022). The absence of independent replication and validation in cervical cancer limits the generalizability of these findings. Other studies concerning mitochondrial morphology, mass, membrane potential, and ATP synthesis also derive from non-cervical systems (Akinbiyi et al., 2021; Jóźwiak et al., 2021) (Table 1). Cervical cancer studies should combine the manipulation of OGT/OGA or candidate substrates with direct measurements of respiration, ATP, ROS, membrane potential, and apoptosis instead of relying on global O-GlcNAc levels alone.

Mitochondrial dynamics provide a representative example of this evidence gap. Drp1 is a key mediator of mitochondrial fission (Chen et al., 2023; Lin et al., 2020), and O-GlcNAcylation of Drp1 has been demonstrated in non-cervical models, where it is associated with mitochondrial recruitment and increased fission (Akinbiyi et al., 2021; Park et al., 2021) (Table 1). However, direct Drp1 O-GlcNAcylation, the relevant modification sites, and its functional consequences have not been established in HPV-positive cervical cancer. Drp1 should therefore be regarded as a cross-model mechanistic candidate rather than a validated cervical cancer O-GlcNAcylation substrate.

OPA1 is essential for inner-membrane fusion and cristae stability (Giacomello et al., 2020; Fry et al., 2024), and altered cristae architecture can influence respiratory-chain organization, cytochrome c storage, and apoptosis initiation (Kawano et al., 2023). Changes in O-GlcNAcylation status have been associated with mitochondrial phenotypes involving OPA1 in other systems, but current evidence is insufficient to establish OPA1 as a direct O-GlcNAcylation substrate (Akinbiyi et al., 2021; Kawano et al., 2023). In this review, OPA1 is therefore treated as a functional marker of inner-membrane remodeling rather than as a confirmed O-GlcNAcylation target in cervical cancer.

The proposed effect of O-GlcNAcylation on mitochondrial apoptosis also remains incompletely defined in cervical cancer. A fall in membrane potential, increased outer-membrane permeabilization, and cytochrome c release can activate the caspase cascade. Studies in other models link altered O-GlcNAcylation to mitochondrial apoptotic processes (Jóźwiak et al., 2021; Xue et al., 2024), but direct causal evidence in HPV-positive cervical cancer is insufficient. Future validation should therefore test whether manipulating OGT/OGA or a defined O-GlcNAcylated substrate changes respiratory chain function, ATP, ROS, membrane potential, Mitochondrial morphology involving Drp1/OPA1, cytochrome c release, and treatment response in cervical cancer models.

### 4.2 Metabolic routes that could indirectly affect mitochondrial adaptation

The HK2/VDAC1 complex at the outer mitochondrial membrane links glycolysis to apoptosis control (Shoshan-Barmatz et al., 2020; Guo et al., 2023). In non-small cell lung cancer, HK2 O-GlcNAcylation was reported to regulate HK2 activity, mitochondrial dynamics, proliferation, and apoptosis (Panpan et al., 2025) (Table 1). In ovarian cancer, the HSP70/HK2/VDAC1 pathway was associated with cisplatin resistance (Li et al., 2021). Whether the two mechanisms converge in cervical cancer remains untested (Very and El Yazidi-Belkoura, 2022).

In non-cervical models, O-GlcNAcylation of PFK1 was reported to alter glucose partitioning, with a potential increase in NADPH production and antioxidant capacity through the PPP (Campos and Albrecht, 2023; Liu et al., 2020; Yi et al., 2012) (Table 1). GFAT1 is also implicated in feedback between HBP activity and glucose utilization (Paneque et al., 2023; Ruegenberg et al., 2020; Yang et al., 2025) (Table 1). The PFK1 and GFAT1 findings identify a testable indirect route by which O-GlcNAcylation could alter mitochondrial redox balance through glucose partitioning without directly modifying mitochondrial proteins. The relevance of this indirect route to ROS regulation and treatment response in HPV-positive cervical cancer remains to be established. Figure 3 summarizes these candidate mitochondrial mechanisms.

**FIGURE 3:**
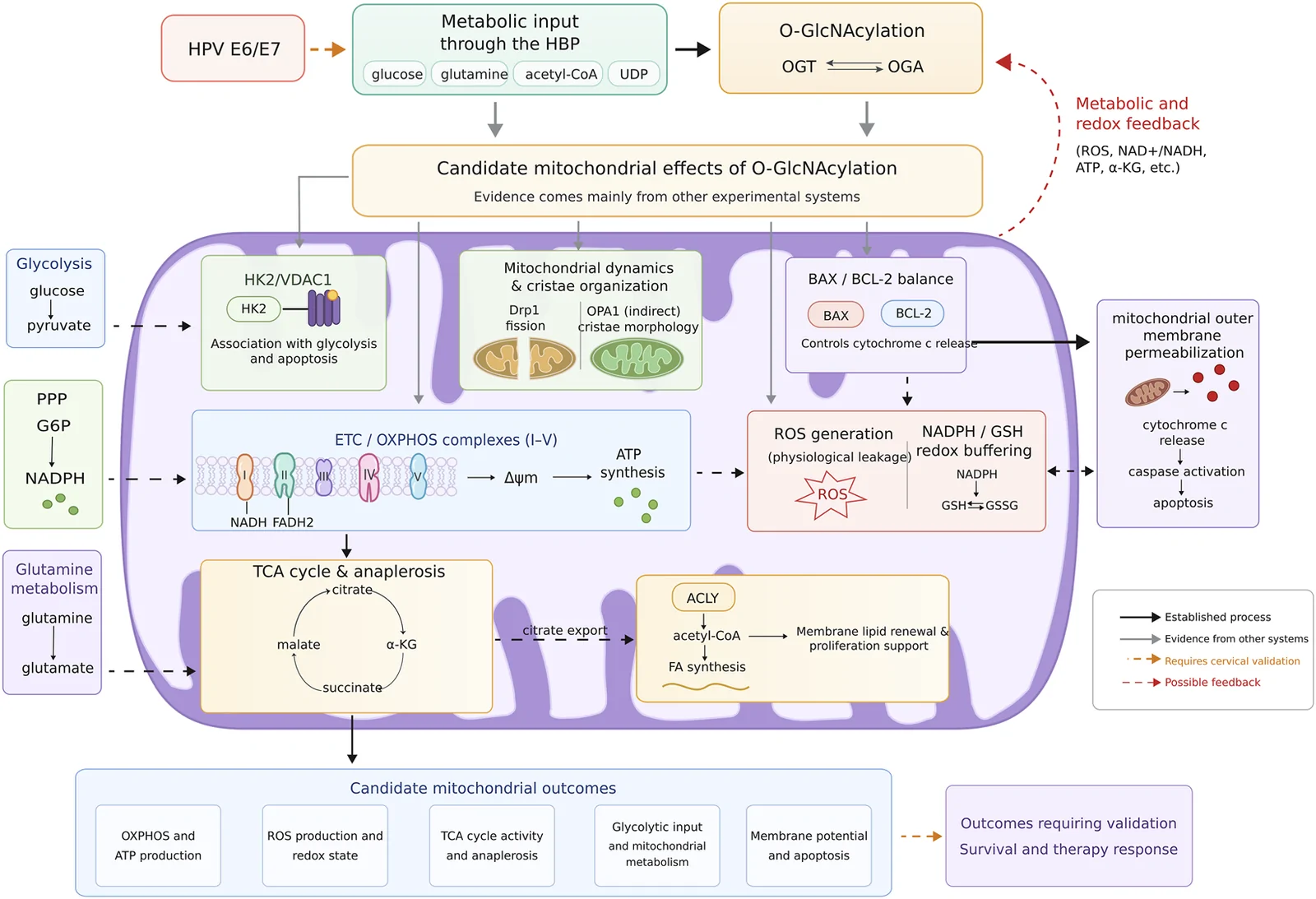
Candidate mitochondrial effects of O-GlcNAcylation in cervical cancer. The schematic includes glycolysis, the pentose phosphate pathway, glutamine metabolism, mitochondrial dynamics, OXPHOS, TCA cycle metabolism, redox regulation, lipid synthesis, and apoptosis. Black solid arrows indicate established processes. Gray solid arrows indicate evidence from other experimental systems. Orange dashed arrows indicate relationships requiring validation in cervical cancer. Red dashed arrows indicate possible feedback. Specific mitochondrial O-GlcNAcylation sites have not been functionally validated in cervical cancer models expressing HPV E6/E7.

## 5 O-GlcNAcylation and the cervical cancer microenvironment

Cervical tumors are characterized by hypoxia, lactate accumulation, inflammatory signaling, and nutrient fluctuation (Zhang Z. et al., 2025; Li et al., 2023). However, evidence from cervical cancer for O-GlcNAcylation in individual cell types within the TME remains limited. Current evidence includes tumor-cell OGT/STAT1 perturbation in an *in vitro* cervical cancer coculture model (Wu et al., 2026), whereas O-GlcNAcylation within T cells, macrophages, and CAFs is inferred mainly from other experimental systems (Zhao et al., 2024; He et al., 2023) (Table 1).

In cervical cancer, tumor-cell lactate accumulation has been linked to immune escape (Xiong et al., 2024). Evidence from general immunological models (Chang et al., 2020; Qiang et al., 2021; Cai et al., 2023; Ramakrishnan, 2024) further supports testing whether nutrient competition, lactate accumulation, and hypoxia alter O-GlcNAcylation in tumor-infiltrating T cells. The proposed relationship requires direct testing in phenotypically defined, spatially resolved, and longitudinally sampled T cell populations.

Regarding macrophages, cervical cancer-specific evidence shows that lactate secreted by cervical cancer cells can modulate macrophage cytokine production and promote a suppressive phenotype in spheroid coculture models (Stone et al., 2019). Evidence from other models indicates that tumor-derived lactate can promote M2-like macrophage polarization (Vadevoo et al., 2021). Chronically elevated macrophage O-GlcNAcylation has been linked to altered inflammatory programs (Abramowitz and Hanover, 2022), whereas hyperglycemia-driven increases in TAM O-GlcNAcylation can favor immunosuppressive polarization (Rodrigues Mantuano et al., 2020). A separate cross-model study identified lysosomal Cathepsin B as an O-GlcNAcylated substrate in TAMs and linked this modification to cancer metastasis and chemoresistance (Shi Q. et al., 2022). A recent cervical cancer coculture study showed that OGT directly O-GlcNAcylated signal transducer and activator of transcription 1 (STAT1) at Ser727 in CaSki tumor cells. OGT overexpression and depletion, STAT1 knockout, and Ser727 mutation altered macrophage polarization and phagocytic activity (Wu et al., 2026). However, the study did not examine O-GlcNAcylation within macrophages. Independent replication, *in vivo* validation, and confirmation in patient-derived samples remain necessary.

Single-cell RNA sequencing of five cervical cancer tissues identified distinct CAF subgroups and associated an extracellular-matrix CAF signature with tumor-promoting and prognostic features (Wang Y. et al., 2024), but this study did not examine HBP flux or O-GlcNAcylation. Evidence linking hypoxia to CAF remodeling derives mainly from general tumor models (Yang et al., 2023; Kim et al., 2022), whereas evidence that O-GlcNAcylation regulates myofibroblast activation and matrix production comes from non-cancer fibrosis models (Very et al., 2024). Whether OGT/OGA activity or defined O-GlcNAcylated substrates regulate CAF activation, matrix remodeling, or metabolic exchange in cervical cancer therefore remains unknown. Together, the tumor and fibrosis studies support the biologically plausible and testable hypothesis that O-GlcNAcylation links metabolic stress to CAF activation and matrix remodeling in cervical cancer.

## 6 Evidence hierarchy and validation boundaries

### 6.1 Classification of O-GlcNAcylation evidence and temporal context

To avoid conflating association with causality, we assess each reported relationship by disease context, causal depth, and temporal context. Functional evidence requires a specific perturbation linked to a measurable phenotype. Evidence for a specific modification site additionally requires validation of the modified substrate and residue (Moon et al., 2022; Griffin et al., 2026). Associations without causal perturbation are classified separately. Evidence from other models is used to assess mechanistic plausibility. The corresponding tiers are defined in Table 1.

Because changes in O-GlcNAcylation depend on the type of stress and the time of measurement (Xue et al., 2024), we report the experimental timing used in each study. For treatment studies, we record the exposure time, whether the cells had been exposed to the drug before, and whether a resistant model was used. Results after a single drug exposure are described as treatment responses at the reported time point. Such results are not considered evidence of acquired resistance. In cell models, acquired resistance requires repeated drug selection from a sensitive parental line and confirmation that the selected cells are less sensitive than the parental line. Whether resistance remains after drug withdrawal should also be tested where feasible (Liu et al., 2018; Ravindran Menon et al., 2023; Pendleton et al., 2023). Measurements made in untreated HPV-positive models show only what was present when the samples were collected. They do not show that the observed findings developed during persistent HPV infection.

Under the criteria defined above, two reported relationships meet the Tier 1A criteria in cervical cancer models. Site-directed mutation of NF-κB p65 Thr322 or Thr352 in HeLa cells reduced CXCR4 expression (Ali et al., 2017) (Table 1). In CaSki cells, OGT overexpression and depletion, STAT1 knockout, and mutation of Ser727 supported a role for tumor-cell STAT1 O-GlcNAcylation in macrophage polarization and phagocytosis in coculture (Wu et al., 2026) (Table 1). The study of circVPRBP and RACK1 provides functional evidence in cervical cancer cells, but the RACK1 S122A mutation was tested in HEK293T cells (Zhang et al., 2023) (Table 1). It is therefore classified as Tier 1B. None of these proteins is mitochondrial.

Other cervical cancer studies provide functional or pathway evidence without a validated causal O-GlcNAcylation site. Proteomic analysis of the OGT interactome linked OGT-associated networks to epithelial-mesenchymal transition and metastasis in cervical cancer (Gao et al., 2018) (Table 1). GFPT1 promoted proliferation through regulation of phosphatase and tensin homolog (PTEN) stability (Li et al., 2022) (Table 1). OGT enhanced secretory clusterin expression through liver X receptor (LXR) and sterol regulatory element-binding protein (SREBP) signaling. Stable OGT depletion also increased cisplatin sensitivity after 24 h in HeLa cells (Kim M. J. et al., 2018) (Table 1). Because OGT depletion had been established before cisplatin treatment, the experiment did not test whether cisplatin induced a protective O-GlcNAcylation response. Metformin treatment for 24 h was associated with reduced AMP-activated protein kinase (AMPK) O-GlcNAcylation, cell cycle arrest, and apoptosis in HeLa cells (Kim et al., 2019) (Table 1).

Together, the cervical cancer literature links HBP activity and O-GlcNAcylation with proliferation, metastasis, immune behavior, and responses during drug treatment. Work in other models identifies candidate mechanisms involving mitochondrial metabolism and dynamics. However, no investigation has combined findings from cervical cancer with direct validation of a mitochondrial O-GlcNAcylation substrate or site. The cisplatin and metformin findings are described as responses at 24 h because neither study used a validated resistant model (Zhao et al., 2010; Lõhmussaar et al., 2021). Table 1 maps the available evidence according to disease model, causal depth, perturbation and timing, mitochondrial evidence, and reported outcome. It therefore identifies candidate mechanisms and specific evidence gaps for future study. The principal evidence gap is the absence of site-specific validation of candidate mitochondrial proteins in HPV-positive cervical cancer, together with direct measurements of respiration, ROS, membrane potential, and mitochondrial dynamics.

### 6.2 Translational implications of the proposed mechanistic framework

Current evidence on O-GlcNAcylation in cervical cancer remains fragmented. Most reports examine global OGT or OGA manipulation or isolated signaling proteins. Treatment experiments capture only short-term responses, and candidate mitochondrial substrates and modification sites derive largely from other models (Table 1). The main gap is therefore not a shortage of candidate targets but the absence of a cervical cancer-specific causal framework. We propose a testable relationship among HPV E6/E7-associated metabolic demand, HBP activity, and substrate-specific O-GlcNAcylation, with potential consequences for mitochondrial and immune adaptation and treatment response. Current evidence does not establish this complete causal sequence but supports testing each component in cervical cancer.

Stable OGT depletion increased cisplatin sensitivity after 24 h in HeLa cells (Kim M. J. et al., 2018) but did not establish that cisplatin induced a protective O-GlcNAcylation response or acquired resistance. Candidate mitochondrial mechanisms require direct validation in cervical cancer models expressing HPV E6/E7, whereas STAT1 Ser727 O-GlcNAcylation in CaSki tumor cells altered macrophage behavior in coculture without demonstrating O-GlcNAcylation within immune cells (Wu et al., 2026). Accordingly, translational studies should validate the functional effects of defined substrates and modification sites in specific HPV and treatment contexts before considering global OGT/OGA inhibition (Very and El Yazidi-Belkoura, 2022; Zhang et al., 2024). Defined substrates or modification sites could ultimately support biomarkers or selective combination strategies, but only after causal validation (Hayes, 2021; Ou et al., 2021) and confirmation in models derived from patients and *in vivo* models (Lõhmussaar et al., 2021; Kutle et al., 2023). Potential interventions must also be evaluated in normal cervical epithelial and immune cells to establish tumor selectivity, define the therapeutic window, and exclude impairment of antitumor immunity (Qiang et al., 2021; Stine et al., 2022). Biomarker and treatment applications therefore remain early preclinical or conceptual. Figure 4 outlines the proposed validation framework.

**FIGURE 4:**
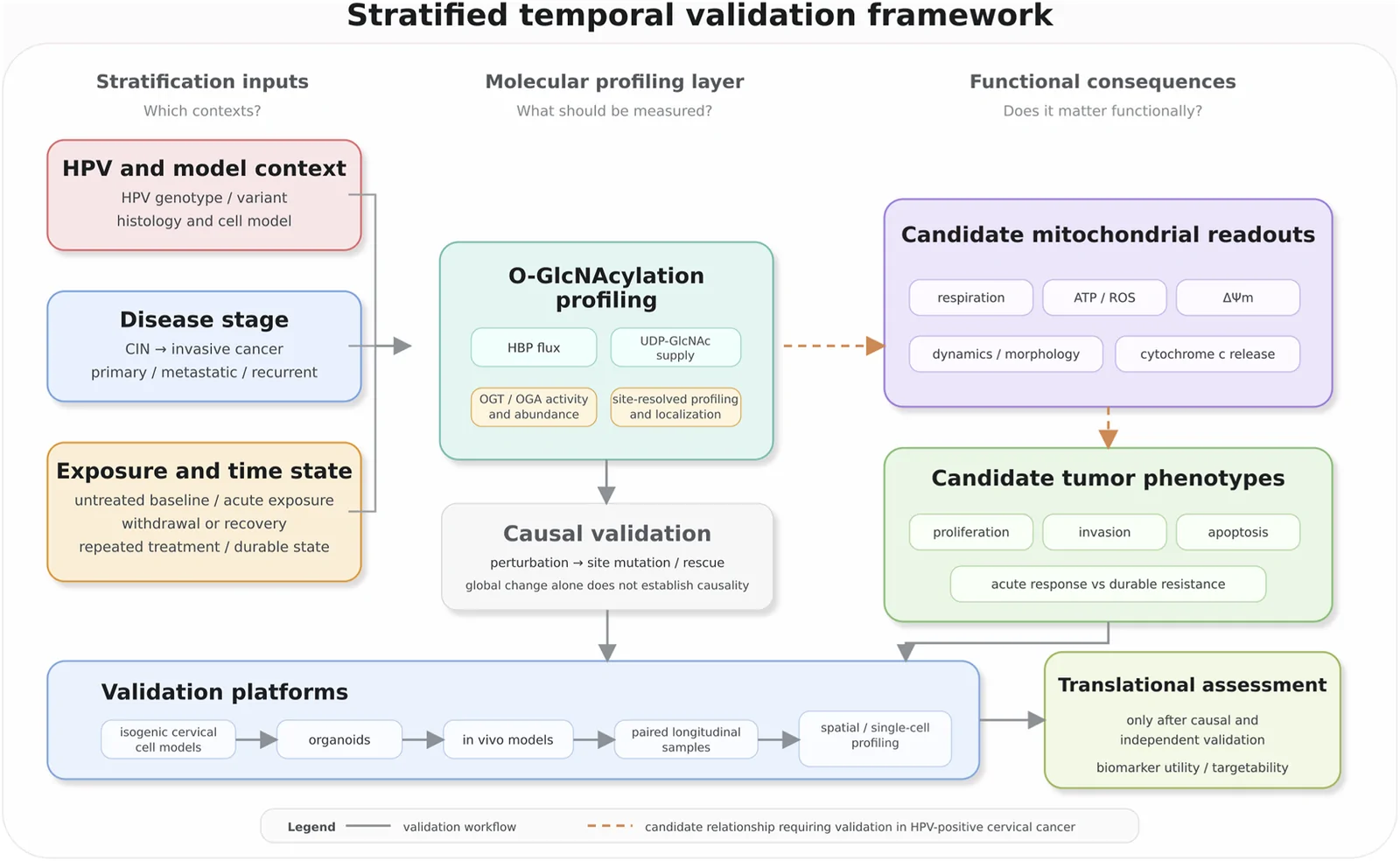
Stratified temporal validation framework for O-GlcNAcylation in cervical cancer. Experiments should stratify HPV genotype or variant, histology, disease stage, and exposure history; distinguish the untreated steady state, acute treatment response, recovery after treatment withdrawal, and established resistance; and integrate O-GlcNAcylation profiling with mitochondrial readouts, persistence testing, causal rescue, tumor phenotypes, and patient-derived mode.

## 7 Conclusion

We propose an integrated and testable mechanistic framework in which sustained HPV E6/E7 activity increases metabolic and biosynthetic demand. The framework treats changes in nutrient use and cellular stress as potential regulators of HBP substrate availability and the O-GlcNAcylation of selected proteins. Altered O-GlcNAcylation could affect signaling and metabolic processes that regulate mitochondrial energy production, redox control, dynamics, and apoptosis. The model complements the established effects of E6/E7 on p53 and pRB rather than replacing them and accommodates variation among cervical cancers. HPV genotype, histology, cell type, nutrient availability, hypoxia, lactate accumulation, and exposure history are therefore considered candidate modifiers of O-GlcNAcylation patterns and the resulting cellular phenotype.

Current evidence supports individual components of this framework, but not the complete causal sequence in HPV-positive cervical cancer; the integrated framework therefore remains partly hypothetical. Direct cervical cancer evidence is strongest for selected non-mitochondrial substrates and signaling effects. Mitochondrial substrates, modification sites, and functional consequences require direct functional validation in HPV-positive cervical cancer models and confirmation in patient derived samples. Studies combining site-specific perturbation with measurements of HBP flux, respiration, ROS, mitochondrial dynamics, and apoptosis will be needed to test the proposed mechanism. Treatment response and durable resistance are later applications of this framework and should be evaluated only after the underlying mechanism has been established. O-GlcNAcylation is therefore positioned as a context-dependent regulatory layer within a broader PTM network, rather than as the sole initiator of metabolic reprogramming.

## Glossary

HPV: human papillomavirus
O-GlcNAc: O-linked β-N-acetylglucosamine
PTM: post-translational modification
OGT: O-GlcNAc transferase
OGA: O-GlcNAcase
UDP-GlcNAc: uridine diphosphate N-acetylglucosamine
HBP: hexosamine biosynthetic pathway
ATP: adenosine triphosphate
ROS: reactive oxygen species
OXPHOS: oxidative phosphorylation
TCA: tricarboxylic acid cycle
HPV16: human papillomavirus type 16
HPV18: human papillomavirus type 18
E6AP: E6-associated protein
HIF-1α: hypoxia-inducible factor 1α
PI3K: phosphoinositide 3-kinase
mTOR: mechanistic target of rapamycin
NF-κB: nuclear factor κB
HCF-1: host cell factor 1
mTORC1: mechanistic target of rapamycin complex 1
Drp1: dynamin-related protein 1
OPA1: optic atrophy 1
HK2: hexokinase 2
VDAC1: voltage-dependent anion channel 1
PKM2: pyruvate kinase M2
UTP: uridine triphosphate
GFAT1: glutamine:fructose-6-phosphate amidotransferase 1
IGF1R: insulin-like growth factor 1 receptor
LDHA: lactate dehydrogenase A
PFK1: phosphofructokinase 1
PPP: pentose phosphate pathway
NADPH: reduced nicotinamide adenine dinucleotide phosphate
TME: tumor microenvironment
STAT1: signal transducer and activator of transcription 1
CAF: cancer-associated fibroblast
TAM: tumor-associated macrophage
RACK1: receptor for activated C kinase 1
GFPT1: glutamine-fructose-6-phosphate transaminase 1
PTEN: phosphatase and tensin homolog
AMPK: AMP-activated protein kinase
sCLU: secretory clusterin
ACLY: ATP citrate lyase
ETC: electron transport chain
ΔΨm: mitochondrial membrane potential
GSH: reduced glutathione
PDK1: pyruvate dehydrogenase kinase 1
PDK2: pyruvate dehydrogenase kinase 2
CKS2: CDC28 protein kinase regulatory subunit 2
SSBP1: single-stranded DNA-binding protein 1
NAD: nicotinamide adenine dinucleotide
NADH: reduced nicotinamide adenine dinucleotide
CXCR4: C-X-C motif chemokine receptor 4
LXR: liver X receptor
SREBP: sterol regulatory element-binding protein
